# Non-invasive ventilation in the care of patients with chronic obstructive pulmonary disease with palliative care needs: a scoping review

**DOI:** 10.1186/s12904-024-01365-y

**Published:** 2024-01-29

**Authors:** Simen A. Steindal, Kristin Hofsø, Hanne Aagaard, Kari L. Mariussen, Brith Andresen, Vivi L. Christensen, Kristin Heggdal, Marte-Marie Wallander Karlsen, Monica E. Kvande, Nina M. Kynø, Anne Kathrine Langerud, Mari Oma Ohnstad, Kari Sørensen, Marie Hamilton Larsen

**Affiliations:** 1 Lovisenberg Diaconal University College, Lovisenberggt 15B, 0456 Oslo, Norway; 2 Faculty of Health Sciences, VID Specialized University, Mail Box 184 Vinderen, 0319 Oslo, Norway; 3 Department of Research and Development, Division of Emergencies and Critical Care, Oslo University Hospital, Oslo, Norway; 4 The Department of Cardiothoracic Surgery, Oslo University Hospital, Oslo, Norway; 5 University of South-Eastern Norway, Drammen, Norway; 6 Department of Nursing and Health Promotion, Acute and Critical Illness, Oslo Metropolitan University, Oslo, Norway; 7 Department of Pediatric and Adolescent Medicine, Division of Neonatal Intensive Care, Oslo University Hospital, Oslo, Norway; 8 Department of Post-Operative and Critical Care, Division of Emergencies and Critical Care, Oslo University Hospital, Oslo, Norway; 9 Department of Pain Management and Research, Division of Emergencies and Critical Care, Oslo University Hospital, Oslo, Norway

**Keywords:** End-of-life care, Non-invasive ventilation, Palliative care, Pulmonary Disease, Chronic Obstructive, Review

## Abstract

**Background:**

Patients with severe chronic obstructive pulmonary disease (COPD) could have palliative care (PC) needs because of unmet needs such as dyspnoea. This may lead to anxiety and may have an impact on patients’ ability to perform daily activities of living. PC can be started when patients with COPD have unmet needs and can be provided alongside disease-modifying therapies. Non-invasive ventilation (NIV) could be an important measure to manage dyspnoea in patients with COPD in need of PC. A scoping review was conducted to gain an overview of the existing research and to identify knowledge gaps. The aim of this scoping review was to systematically map published studies on the use of NIV in patients with COPD with PC needs, including the perspectives and experiences of patients, families, and healthcare professionals (HCPs).

**Methods:**

This review was conducted following the framework of Arksey and O’Malley. The reporting of the review was guided by the Preferred Reporting Items for Systematic Reviews and Meta-Analyses extension for Scoping Reviews checklist. The review protocol was published. AMED, CINAHL, Embase, MEDLINE, PEDro, and PsycInfo were searched from inception to November 14, 2022. The included studies had to report the perspectives and experiences of COPD patients, relatives, and HCPs regarding NIV in the care of patients with COPD with PC needs. In pairs, the authors independently assessed studies’ eligibility and extracted data. The data were organised thematically. The results were discussed in a consultation exercise.

**Results:**

This review included 33 papers from 32 studies. Four thematic groupings were identified: preferences and attitudes towards the use of NIV; patient participation in the decision-making process of NIV treatment; conflicting results on the perceived benefits and burdens of treatment; and heterogenous clinical outcomes in experimental studies. Patients perceived NIV as a ‘life buoy’ to keep them alive. Many patients wanted to take part in the decision-making process regarding NIV treatment but expressed varying degrees of inclusion by HCPs in such decision-making. Conflicting findings were identified regarding the perceived benefits and burdens of NIV treatment. Diversity in heterogeneous clinical outcomes were reported in experimental studies.

**Conclusions:**

There is a need for more studies designed to investigate the effectiveness of NIV as a palliative measure for patients with COPD with PC needs using comprehensive outcomes. It is especially important to gain more knowledge on the experiences of all stakeholders in the use of home-based NIV treatment to these patients.

**Supplementary Information:**

The online version contains supplementary material available at 10.1186/s12904-024-01365-y.

## Background

The scope of palliative care (PC) has broadened in the last decade to include care of patients with a life-limiting disease, such as chronic obstructive pulmonary disease (COPD) [1–3]. PC is recommended to be initiated early in the disease trajectory [4]; however, as there is no accepted method for predicting prognosis or defining end-stage COPD [5], it is difficult to initiate PC [6]. Consequently, PC should instead be initiated on the basis of refractory symptoms, patients’ preferences, physical, psychological, social or spiritual/existential unmet needs [7, 8]. According to a recent task force report from the European Respiratory Society, PC in patients with COPD should start when an unmet need arises [8]. PC can be provided to these patients alongside disease-modifying therapies [9].

Dyspnoea is the most frequent and burdensome symptom in patients with severe COPD and may impact patients’ ability to perform daily activities, such as dressing, walking, and eating [10–12]. Experiencing dyspnoea often leads to anxiety and panic as well as concern about death and dying [10, 13].

Non-invasive ventilation (NIV) is the standard treatment for patients admitted to hospitals with COPD exacerbation and acute respiratory failure [14]. NIV may be appropriate for managing severe dyspnoea by improving ventilation, oxygenation, and the resistive load on the ventilatory muscles, all of which reduce the work of breathing [14–16]. In recent decades, a substantial increase in the use of NIV has been found among older hospitalised patients with terminal respiratory illness, suggesting a major shift in the way healthcare professionals (HCPs) provide ventilatory support in the palliative phase [17]. NIV seems to be used in around 33% of patients with COPD with poor life expectancy [18]. Home-based NIV is frequently used in patients with COPD and chronic hypercapnic respiratory failure [16]. Physicians involved in home-based NIV have reported that the main expected benefits of NIV treatment for patients with COPD are alleviation of dyspnoea, improvement in quality of life (QOL), and a reduction in the number of hospitalisations [19]. Alleviation of dyspnoea is considered important when NIV is used as life support for patients who have decided to forgo invasive mechanical ventilation (IMV) or as a palliative measure [20]. However, the use of NIV as a palliative measure is controversial. NIV could improve QOL and provide comfort, however, NIV could also be a futile treatment that may prolong the dying process without improving QOL [21, 22]. Concerns have been raised by HCPs as to whether patients and relatives fully understand the goals of care when NIV is used as a PC measure [23]. In this paper, patients with severe or very severe COPD undergoing NIV treatment are understood as patients with PC needs.

Previous reviews and meta-analyses have explored the impact of home-based NIV on clinical outcomes in patients with COPD [24, 25], including its effect on mortality in acute settings [26], and addressing dyspnoea during acute exacerbations, and respiratory failure [27].

One systematic review and meta-analysis investigated NIV use across various diagnosis, encompassing acute respiratory failure, with do-not-intubate or comfort-measures-only orders [28]. An integrated review found that combining NIV with long-term oxygen treatment (LTOT) may alleviate hypercapnia and dyspnea in patients with COPD [29]. However, most studies on NIV have focused on avoiding intubation in end-stage COPD rather than providing symptom relief [29]. Another systematic review on NIV in PC suggested potential improvements in QOL and dyspnoea for end-stage lung disease patients [30].

Previous reviews of the use of NIV in patients with PC needs have not solely focused on patients with COPD and have included only quantitative studies. In our initial search of the literature, we did not find any scoping review that had mapped studies on the use of NIV in patients with COPD with PC needs. Conducting a scoping review that includes various research methods and study designs, as well as the experiences and perspectives of patients, relatives, and HCPs, is important to achieve a more comprehensive understanding [31]. Such a review could inform clinical practice by summarizing exiting evidence regarding the use of NIV to these patients. Furthermore, our review may identify research gaps that are important to develop interventions to improve patients’ compliance to NIV, improve the effectiveness of NIV, and to prioritize future research in this field. This scoping review aimed to systematically map published studies on the use of NIV to patients with COPD with PC needs. Our research question was: What is known about the use of NIV from the perspectives and experiences of patients with COPD with PC needs, their relatives, and HCPs?

## Methods

This scoping review employed the methodological framework described by Arksey and O’Malley [31]. We performed the optional stage consultation exercise to make the findings more useful and relevant for clinical practice [31]. The reporting of the review was guided by the Preferred Reporting Items for Systematic Reviews and Meta-Analyses (PRISMA) extension for Scoping Reviews checklist [32]. Deviations from the published protocol [33] are described in Additional file 1.

### Eligibility criteria

We used the Sample, Phenomenon of Interest, Design, Evaluation, and Research Type (SPIDER) framework [34] to describe the eligibility criteria (Table 1).

**Table 1:** Eligibility criteria using the SPIDER framework

	**Inclusion**	**Exclusion**
Sample (S)	Papers including patients with severe or very severe COPD aged 18 years or older in need of PC, including advanced, late-stage, and end-stage COPD and do-not-resuscitate or comfort-measures-only orders. Patients with severe or very severe COPD using NIV treatment are defined as patients with PC needs Papers including relatives of COPD patients in need of PC Papers including HCPs caring for COPD patients with PC needs Studies will be included regardless of reasons for or length of NIV treatment	Papers including patients younger than 18 years and patients that have mild and moderate COPD Papers including relatives of patients with mild or moderate COPD Papers including HCPs caring for patients with mild to moderate COPD Papers including patients without a COPD diagnosis
Phenomenon of Interest (PI)	Studies related to NIV treatment, including mask, intermittent positive-pressure ventilation, bilevel positive airway pressure, and continuous positive airway pressure, in all healthcare settings and in all phases of the PC trajectory. Studies will be included if the COPD patients have been treated with NIV in PC, regardless of the reasons for or length of NIV treatment Use of NIV to prolong life and/or for alleviation and comfort	Use of NIV with a curative intention Studies exploring other respiratory interventions or treatment exercises and studies focusing on other palliative measures Studies including diseases other than COPD
Design (D)	Studies with qualitative, quantitative, or mixed-methods designs	
Evaluation (E)	Perspectives and experiences of COPD patients, relatives, and HCPs regarding NIV to patients with COPD with PC needs	Studies exploring the experiences and perspectives of students Studies investigating patients’, relatives’, or HCPs’ experiences of specific interventions or treatments
Research Type (R)	All research types of peer-reviewed studies published in scientific journals in German, Spanish, Swedish, Danish, Norwegian, or English	Case studies, case–control studies, reviews of any type, clinical guidelines, and master’s and PhD theses Grey literature, such as conference proceedings and abstracts, letters, comments, editorials, and non-peer-reviewed papers

### Information sources

We searched AMED, CINAHL, Embase, MEDLINE, PEDro, and PsycInfo from inception to February 1, 2020 to identify relevant studies published in peer-reviewed journals. The database search was updated on November 14, 2022. We set no limit on the year of publication, as we wanted to describe the entire range of published studies relevant for our research question [35].

### Search

The fourth author, an experienced librarian, and the first and last author built a comprehensive and systematic search strategy in MEDLINE using medical subject headings and text words and the search strategy was discussed with the other authors and tested. The final search strategy in MEDLINE was adapted for the other databases, which were peer-reviewed by a second librarian using the Peer Review of Electronic Search Strategies checklist [36]. The search strategy for all the databases is described in Additional file 2. The database search was limited to papers published in Danish, English, German, Norwegian, Spanish, and Swedish since we speak these languages fluently. We applied publication type filters to exclude editorials, letters, comments, and conference abstracts according to the functionality of each database to ensure that only studies published in peer-reviewed journals were included.

We performed manual searches to screen the reference lists of the included papers.

### Selection of the sources of evidence

The fourth author transferred the search results to EndNote for duplicate removal and then transferred the search results to Covidence (covidence.org), a web-based systematic review application, to facilitate storage and blinding of the study selection process. Covidence ensured that two authors independently assessed whether titles and abstracts and then full-text papers met the inclusion criteria (Table 1). In cases of disagreement, two authors (SAS, MHL) performed an independent assessment, and a final decision was based on consensus between these two authors.

### Data charting

We developed a standardised data-charting form in Covidence using the SPIDER framework. The data-charting form was piloted by two pairs of authors who independently extracted data from five of the included papers. After the pilot we removed research type (R) from the data-charting form as it overlapped with design (D). The final data-charting form included the following information: author, year of publication, country, setting, sample; aim; design; findings. One author extracted data, while another checked the accuracy of the extracted data against the papers. In cases of disagreement, a third author (SAS, MHL) independently extracted data and made the final decision.

### Synthesis of results

We inductively summarised and organised the data thematically [31]. First, we extracted NIV-related findings from the results section of the included studies, and these data were read several times to identify patterns of similarities and differences in perspectives and experiences regarding the use of NIV to patients with COPD with PC needs across the included studies. We sorted text related to the identified patterns into thematic groups. The first and last author analysed the data, while all the authors discussed the emerging patterns and agreed upon the final thematic grouping [37–39].

### Consultation exercise

To enhance the review findings’ relevance for clinical practice, we conducted a consultation exercise [31]. We recruited respiratory nurses and critical care nurses to an advisory board through information posted on the university college’s web page and on social media (i.e., Facebook and Instagram) as well as asking key persons in the respiratory nursing and critical care nursing community to share information about the consultation exercise in their networks. Five nurses contacted the first author and wanted to participate. The characteristics of the participants are described in Table 2.

**Table 2:** Description of the participants

***N* = 5**	
Respiratory ward	3
High dependency unit (medical)	2
Male	2
Age, median (range)	39 (27–48)
Years of experience in lung ward or high dependency unit (medical), median (range)	19 (3–22)
Bachelor of Science in nursing	5
Postgraduate education	
Respiratory nursing	2
Critical care nursing	1
Master’s degree in critical care nursing	1

We facilitated four one-hour workshops using videoconferencing (Zoom); three workshops consisted of one participant in each workshop, while two participants participated in one workshop. The first and second author attended and presented the findings related to the thematic groupings (one acted as moderator and the other as secretary). We used an interview guide to facilitate reflection and dialogue with the participants regarding which of the review findings were most important, relevance for clinical practice, whether the findings were surprising or expected, findings that were missing, and what future research regarding NIV in PC by patients with COPD should address. The audio recording from the videoconference was transcribed verbatim by an external transcriber.

### Ethical considerations

We attained approval from the Norwegian Centre for Research Data (reference number 480222) before we conducted the workshop with the advisory board. The information highlighted that participation was voluntary and that anonymity and confidentiality would be safeguarded. The participants signed informed written consent.

## Results

### Study selection

**Fig. 1:**
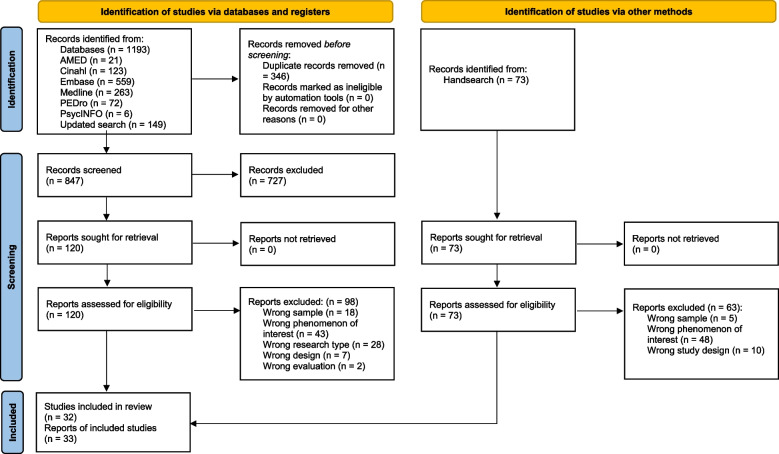
PRISMA flow diagram of the search and screening process

The database searches yielded 1193 publications. After 346 duplicates were removed, titles and abstracts for 847 publications were screened. Based on the inclusion and exclusion criteria, the full text of 120 publications was read, and 23 studies were included. The manual search identified nine studies from 10 papers. The reasons for the exclusion of full-text papers are presented in Fig. 1.

### Study characteristics

In total, 33 papers from 32 studies were included. The studies were published between 1992 and 2022, and nine (32%) were published in 2018 or later [40–45]. In total, 17 countries were represented. Six studies were from the United Kingdom [46–51], five from Germany [43, 52–55], three from Norway [44, 56, 57], and two from Austria [41, 58], Italy [59, 60], Taiwan [40, 45], and New Zealand [61, 62], respectively. Furthermore, the following countries were represented with one study each: Australia [63], Belgium [64], Denmark [65, 66] (two papers from the same qualitative study), The Netherlands [67], both the US and Canada [68], Canada [42], Iran [69], Spain [70], France [71], and Brazil [72].

Eight studies had a qualitative design [44, 49, 56, 57, 61, 62, 65, 66], six were randomised controlled trials (RCTs) [43, 58, 63, 70–72], four had a cross-sectional design [41, 42, 48, 68], five were retrospective cohort studies [40, 45, 53, 54, 67], and four cohort studies had a prospective design [47, 51, 52, 59]. In addition, there was one pre-post study [69], one with standardised structured interviews [46], and one follow-up study over two years (pre/post) [50].

A total of 166230 patients with COPD participated in the studies, ranging from 10 to 151912 participants, of whom 81642 (49.1%) were men. Thirty-one studies reported mean age, which was 65.2 years across the studies, ranging from 43 to 83 years.

Five of the studies [48, 49, 57, 65, 66, 68] included 604 HCPs, and two studies included four relatives [49, 65, 66]. The majority of HCPs were different types of physicians (*n* = 131), physicians in training (*n* = 12), general practitioners (*n* = 18), and specialists (n = 132). Other HCPs represented in the studies were respiratory therapists (RT) (*n* = 290), nurses (*n* = 33), physiotherapists (*n* = 2), psychologists (*n* = 2), and health care workers (*n* = 1), and one study did not report occupation.

The majority of the studies were conducted in a hospital setting (*n* = 20), including one outpatient setting [46]; eight studies were home-based [50–53, 55, 58, 60, 69], and two were a combination of home-based and hospital [45, 72]. The other three were from various settings (i.e., pulmonary rehabilitation [43], community centre [68], and population-based [42]).

NIV was used in different situations applicable for patients with COPD. Three studies tested nocturnal NIV plus LTOT [63, 70], one study tested nocturnal NIV [50], and two studies tested NIV during exercise [43, 72]. Other studies investigated NIV as a factor related to cardiopulmonary resuscitation or a do-not-resuscitate order [40, 47, 48]. Additional studies focused on special diseases, such as mustard airway disease [69], or on acute ventilatory support [46]. The characteristics of the included studies are shown in Table 3.

**Table 3:** Characteristics of the included studies

Author, year of publication, and country	Setting	Sample	Aim	Design	Findings
Aliannejad et al. (2015) Iran [69]	Home	20 patients with severe mustard airway; 20 males, mean age 43 (standard deviation (SD) 5) years, mean FEV_1_% predicted 25 (SD 9)	To assess the efficacy of non-invasive ventilation (NIV) in subjects with severe mustard airway disease	One group pre-post study	NIV improved health-related quality of life (HRQOL) in very severe patients. NIV reduced hospitalizations and exacerbations in severe patients. NIV did not improve exercise tolerance, pulmonary function, or dyspnoea
Beckert et al. (2020) New Zealand [62]	Hospital	15 patients with severe or very severe COPD; 9 males, mean age 69.2 (SD 8.2, range 55–89) years, mean FEV_1_% predicted 26.4	To investigate the experiences and perceptions of participants using NIV	Qualitative approach using Grounded theory with inductive coding	NIV was uncomfortable and affected patients’ cognition. It was also considered as a life saver and a concern for others. Patients considered NIV as a viable option for future treatment and described a high level of trust in healthcare professionals and delegated decision-making to them regarding ongoing care
Borghi-Silva et al. (2010) Brazil [72]	Home; university physiotherapy department	14 patients with severe COPD used NIV; 9 males, mean age 68 (SD 9) years, mean FEV_1_% predicted 34 (SD 10) 14 patients with severe COPD used supplemental oxygen; 9 males, age 67 (SD 7) years, mean FEV_1_% predicted 33 (SD 7)	To investigate whether NIV alone could promote a true physiological training effect after training that is greater than that of oxygen supplementation	Randomized controlled trial (RCT)	There were significant differences between the NIV and supplemental-oxygen groups in lactate/speed ratio (33% vs -4%), maximum inspiratory pressure (80% vs 23%), 6-min walk distance (122 m vs 47 m), and leg fatigue (25% vs 11%). Changes in SpO_2_/speed, VO_2_, and dyspnoea were greater with NIV than with supplemental oxygen. HRQOL symptoms and disease impact were significant lowered in both groups. Activity and total St. George Respiratory Questionnaire scores were significantly reduced only in the NIV group
Budweiser et al. (2005) Germany [53]	Home	46 patients with stable COPD undergoing NIV treatment; 38 male, median age 65.2 (range 53.1–77.9) years, median FEV_1_% predicted 29 (SD 8.2)	To evaluate the impact on lung deflation of patients receiving long-term home ventilation, by performing a retrospective analysis of different lung function parameters including inspiratory capacity and respiratory muscle function in a collective of severe symptomatic COPD patients in a stable status of their disease	Retrospective explorative study	One-year survival was 89.1%. There was a significant reduction in nocturnal and daytime PaCO2, a decrease in the ratio of residual volume to total lung capacity at 6 and 12 months. Significant improvements in inspiratory capacity, vital capacity and FEV_1_ were found. For patients with the most severe hyperinflation a significant positive correlation between inspiratory positive airway pressure and reductions in PaCO2 and residual volume/total lung capacity were found
Budweiser et al. (2006) Germany [55]	Home	141 patients in stable state with severe COPD (at baseline); 106 males, median age was 65 (SD 8.4, range 41.7–80.0) years, FEV_1_% predicted 29.7 (SD 9.1%)	To investigate whether initiation of NIV results in an alteration in body weight, particularly in malnourished patients, up to 12 months after initiation of treatment, and whether there is a link to changes in functional variables	Cohort study	Malnutrition (BMI of < 20 kg/m2) was found in 21% of the patients. BMI was significantly correlated with the severity of respiratory impairment, especially with hyperinflation. In malnourished patients there was a significant increase in body weight after 6 months and 12 months, while no significant changes in the overall study population. There was no correlation between changes in BMI and changes in blood-gas values, lung function, or inspiratory muscle function, either in the entire patient group or in the subgroup of malnourished patients
Budweiser et al. (2007) Germany [52]	Home	188 patients with COPD; 147 males, mean age 64.5 (SD 8.0) years, median FEV_1_% predicted 30.0 (SD 9.6)	To focus on predictors of mortality in patients with chronic hypercapnic COPD receiving NIV	Cohort study	The mortality rate during follow-up was 44.7%, with 1-year, 2-year and 5-years survival rates of 84.0%, 65.3% and 26.4%. Death occurs mainly from respiratory causes (73.8%)
Carlucci et al. (2016) Italy [59]	Hospital; respiratory units; 2 rehabilitation centres and 1 respiratory critical care unit	43 patients with very severe COPD; 84% males, median age 72 (interquartile range [IQR] 65, 78) years, 44% use NIV, mean FEV_1_% predicted NR, inclusion criteria: FEV_1_% predicted < 30%	To assess the patients’ preferences regarding end-of-life sustaining interventions, the patient’s ‘comprehension and retention’ of their choices and if at the time of death the patients’ decision was respected	Prospective multicentre study	The choice of NIV a ‘ceiling’ treatment was associated with a current use of NIV and a recent family bereavement. A minority of subjects missed the meaning of ceiling NIV (19%). The wish of patients was respected in about half the patients: all these patients died under mechanical ventilation or NIV. Few relatives reported that patients’ preference changed
Casanova et al. (2000) Spain [70]	Hospital; pulmonary clinics	20 patients with severe COPD in NIV group; 20 males, mean age 64 (SD 65) years, mean FEV_1_% predicted NR 24 patients with severe COPD in control group; 23 males, mean age 68 (SD 4) years, mean FEV_1_% predicted NR	To determine the 1-year efficacy of NIV added to long-term oxygen therapy in patients with stable severe COPD	RCT	One-year survival and the number of acute exacerbations was similar in both groups. The only beneficial differences were observed in the Borg dyspnoea rating, which dropped from 6 to 5 in one of the neuropsychological tests (psychomotor coordination) for the NIV group at 6 months
Chakrabarti et al. (2009) United Kingdom [46]	Outpatient setting	50 patients with COPD patients; 34 males, age 69 (IRQ 14), median FEV_1_% predicted 36	To understand the attitudes of patients with COPD toward acute ventilatory support and assess how aids to decision making regarding ventilation affect patients’ views of therapy	Standardized structured interview	86% found demonstration of NIV helpful in decision making compared to 24% with the photographic aid. 96% were willing to receive NIV after a verbal description of the technique. 76% consented when a photographic aid was shown. When NIV was demonstrated, willingness rose to 84%. Willingness to receive NIV was not significantly associated with gender, domiciliary oxygen use, prior participation in a pulmonary rehabilitation program, social status, whether currently smoking, MRC index, or WHO performance status
Christensen et al. (2017) Denmark [65]	Hospital	16 patients with severe COPD treated with NIV at least once during last 2 years; 6 males, mean age 69 (age range 47–86) years, mean FEV_1_% 24 4 relatives7 HCPs; 3 nurses, 2 physicians, 1 healthcare worker, 1 NR (PhD student, principal researcher)	To investigate user perspectives on health care practice in the hospital concerning NIV treatment; to understand how patients with COPD and health professionals (HCPs) experience and evaluate treatment with NIV; to develop new management strategies for NIV treatment of patients with COPD based on patients’, relatives’ and HCPs’ perspectives on treatment	Qualitative approach using critical psychological practice research	15 patients evaluated treatment with NIV positively, 13 had experienced fear and 14 discomfort during treatment. The co-researcher group described HCPs’ perspectives and analyzed treatment practice based on data from patients’ perspectives developing new management strategies in clinical practice with NIV
Christensen et al. (2018) Denmark [66]	Hospital	16 patients with severe COPD treated with NIV at least once during last 2 years; 6 males, mean age 69 (age range 47–86) years, mean FEV_1_% 24 4 relatives 7 HCPs; 3 nurses, 2 physicians, 1 healthcare worker, 1 NR (PhD student, principal researcher)	To clarify COPD patients’ perspectives on treatment with NIV ventilation and develop management strategies for the treatment based on these perspectives	Qualitative approach using critical psychological practice research	Patients regarded NIV treatment positively even though they experienced discomfort and anxiety. Patients conduct their everyday lives looking at COPD as a basic life condition rather than an illness. This approach had a major impact on patients’ attitudes to NIV treatment and hospitalization
Duenk et al. (2017) The Netherlands [67]	Hospital	33 patients with acute exacerbation of COPD; 19 males, mean age 72 (SD 10.4), mean FEV_1_% predicted NR	To examine whether proactive indicators for palliative care are documented consistently in the medical records and explore the percentage of patients with a poor prognosis and prognostic value	Retrospective medical record review	NIV was always documented as 1 of 10 indicators for palliative care in the hospital setting
Elliott et al. (1992) England [51]	Home	12 patients with severe stable COPD and hypercapnic respiratory failure (HRF); 9 males, mean age 57.4 (SD 5.6) years, FEV_1_% predicted NR	To evaluate the practicalities of nasal intermittent NIV at home in patients with COPD and the effect on sleep and quality of life	Prospective cohort	At 6 months 8 patients were continuing with NIV. At 6 months there was an increase in mean PaO2 of 11% and lower mean transcutaneous carbon dioxide tensions overnight compared with spontaneous breathing before the start of nasal NIV. Total sleep time and sleep efficiency changed during NIV by + 72, 5 min and + 5% respectively. Sleep architecture and the number of arousals were unchanged. QOL did not change but was no worse during NIV. At one year 7 patients were still using NIV and Paco2 and bicarbonate ion concentration during the day had improved further by comparison with the values at six months
Faes et al. (2018) Belgium [64]	Hospital	3872 patients died of COPD; 2597 males, mean age 78.8 (SD 10) years, FEV_1_% predicted NR 19401 patients died with COPD (died of lung cancer or cardiovascular disease); mean age 76.6 (SD NR) years, FEV_1_% predicted NR	To describe EOL resource use in people diagnosed with COPD in the last six months of life and compare this resource use between those dying of COPD, cardiovascular disease, and lung cancer	Retrospective, full- population analysis	Those who died of COPD (51%) were more likely to receive NIV than those who died of cardiovascular disease (22.5%) or lung cancer (37.9%) Those dying of COPD had significantly more days of NIV compared to the other two groups, with a mean of 60.2 days
Fahim & Kastelik (2014) United Kingdom [47]	Hospital	30 patients with COPD for at least 12 months duration; 18 males, mean age 70 (SD 8, age range 43–87) years, mean FEV_1_% predicted 37 (SD 12.8)	To evaluate the COPD patients’ understanding of palliative care as a management option of COPD and to identify any barriers to resuscitation discussion in this group of patients	Prospective observational study	13 patients understood the term NIV. 11 of those would consider it again if needed
Fu et al. (2018) Taiwan [40]	Hospital; admitted for acute care	271 electronic health records from patients with terminal COPD; 249 males, median 83 (IQR 77–88), median FEV_1_% predicted 60.5 (IQR 40–81.8)	To investigate factors associated with an early do-not-resuscitate (DNR) directive	Retrospective observational	Early DNR patients died less frequently in the intensive care unit, received less frequent invasive mechanical ventilation (IMV), more frequent non-invasive MV, and had a shorter length of hospital stay
Funk et al. (2011) Austria [58]	Home	13 patients with COPD in NIV group; 7 males, mean age 62 (SD 6) years, mean FEV_1_% predicted 31 (SD 17) 13 patients with COPD in withdrawal group; 8 males, mean age 65 (SD 6) years, mean FEV_1_% predicted 30 (SD 12)	To determine whether the withdrawal of longterm NIV causes clinical worsening in stable COPD patients who remained hypercapnic after an episode of acute respiratory failure requiring IMV	RCT	After randomisation the withdrawal group had a higher probability of clinical worsening compared to the ventilation group. After 12 months, ten patients in the withdrawal group, but only two patients in the NIV group, experienced a significant clinical worsening. 3 moths after randomization the 6-min walking distance increased in the NIV group and decreased in the withdrawal group
Gaber et al. (2004) United Kingdom [48]	Hospital	100 patients with COPD; 41 males, mean age 74.1 (age range 48–92) years, mean FEV_1_% predicted NR, 44 patients had a FEV1 < 40%, 37 had a FEV1 between 40 and 59% predicted	To ascertain the views of patients with COPD in the community towards artificial ventilation and cardiopulmonary resuscitation (CPR) and whether this sensitive issue could be addressed by respiratory nurse specialists	Survey	48 patients wanted all additional treatments (NIV, IV, CPR) if needed and 12 wanted none. Nineteen patients said ‘no’ for CPR but ‘yes’ to NIV and IV. 10 patients said ‘no’ to CPR and IV but ‘yes’ to NIV. The remaining 11 patients gave other mixed answers. There were no significant differences between the “yes” and “no” group. 98% agreed that this sensitive issue should be discussed with all patients. 1 patient thought that it should be discussed only with seriously ill patients
Gale et al. (2015) UK [49]	Two hospitals	20 patients with COPD (either past or currently use of domiciliary NIV or minimum two episodes of acute NIV use); 8 were male, median age was 68 (age range 52–83) years 4 carers and 15 healthcare professionals (7 doctors, 4 specialist nurses, 2 physiotherapists and 2 physiologists): sex NR, age range 26–54 years	Explore experiences of domiciliary non-invasive ventilation in COPD, to understand decision-making processes and improve future palliative care	Qualitative interview study, based on the constructivist grounded theory	The study identified `adapting to NIV the central process enabling long-term use in palliative care, although the way in which this is approached by HCPs and patients do not always converge. Patients and HCPs actively negotiate the patient's adaption to NIV, although their experiences and views are not always convergent. While domiciliary NIV is valued by COPD patients, The process of adaption could be optimized by HCPs considering broader ways of explaining the process, other settings for initiation and generating more Patient-data on its benefits
Gäbler et al. (2019) Austria [41]	Hospital; ICU, pulmonology internal departments and geriatric/ palliative care	162 physicians (67 ICU, 51 pulmonology or internal departments, 44 geriatric or palliative care); 89 males, mean age 49 (SD 10, age range 27–65) years, 12 were physician in training, 18 general physician, 132 specialist, 110 had ⩾ 10 years of job experience	To investigate if the choice of treatment is influenced by the medical speciality	Cross-sectional survey	38 (23%) respondents chose NIV, 50 (31%) chose conservative treatment approach and 74 (46%) chose palliative approach. Intensivists had an almost 15-fold probability and pulmonologists/internists a nine-fold probability of inducing NIV in comparison with geriatricians/palliative physicians. Increasing age of the physician tended to correlate significantly against starting NIV. No effect was observed due to the following variables: amount (years) of professional experience, educational level and the importance of low patient stress due to the intervention
Gershon et al. (2018) Canada [42]	Population based (Ontario)	151 912 patients with advanced COPD between 2004 to 2014; 47.4% males, 80% were aged > 65 years, mean FEV_1_% predicted NR	To describe trends in the use of EOL care strategies by people with advanced COPD in Ontario, Canada	Repeated cross-sectional study	The proportion admitted to the ICU who needed NIV slightly increased over time. In 2004 around 1% the patients used NIV, while in 2014 around 4% used NIV
Girault et al. (1997) France [71]	Hospital; medical intensive care unit (ICU)	15 patients with known COPD or a high probability of the disease; 12 males, mean age 64.5 (SD 6.75) years, mean FEV_1_% predicted 29.20 (SD 10.43)	To investigate the effects of NIV assisted-control ventilation (ACV) by nasal mask on respiratory physiological parameters and comfort in acute on chronic respiratory failure (ACRF)	RCT	More COPD patients compared with other groups died in the ICU (1.54, p = 0.012). NIV ACV significantly decreased all the total inspiratory work of breathing parameters, pressure time product, and oesophageal pressure variation in comparison with spontaneous breathing (SP) mode. The ACV mode resulted in a significant reduction in surface diaphragmatic electromyographic activity to 36% of the control values and significantly improved the breathing pattern. The respiratory comfort was significantly lower with ACV than with SB
Gloeckl et al. (2019) Germany [43]	Pulmonary rehabilitation	20 patients; 12 males, mean age 60 (SD 6) years, mean FEV_1_% predicted 19 (SD 4)	To investigate the acute effects of high-pressure NIV (along with oxygen supplementation) as an add-on tool during exercise in COPD patients with chronic hypercapnic respiratory failure	RCT, cross-over trial	On NIV COPD patients increased cycle endurance time by 39% compared to oxygen-use only. In NIV condition, TcPCO2 values were significantly lower at rest and at isotime compared to control condition. Oxygen saturation was significantly higher with NIV during exercise. All patients tolerated the use of NIV during exercise well and were able to perform cycle training with NIV. On NIV, TcPCO2 was significantly lower at rest and at isotime. Oxygen availability in the intercostal muscles remained relatively constant with NIV compared to oxygen-use only
Jerpseth et al. (2017) Norway [57]	Hospital; ICU, pulmonary ward	26 nurses (12 ICU and 14 pulmonary ward); 2 males; median age ICU nurses 38 (age range 31–55) years, median age pulmonary ward nurses 34 (age range 25–47), median years of experience ICU 8 (range 1–14) years, median years of experience pulmonary ward 6 years (range 9 months-15 years)	To investigate how nurses experienced their own role in decision-making processes regarding IMV in later stages of COPD and how they consider the patients’ role in these processes	Qualitative design	Nurses described the dilemma of being part of a medical treatment culture rather than being able to focus on the patients’ need for good care at the end of life. This medical culture focused on patients’ capacity to breathe, and the only solution offered to patients was either NIV or IMV. The patients’ situation was so complex that the nurses felt they needed care that extended beyond simply treatment with NIV or IMV. Nurses experienced lack of authority to act; they felt that they should have acted on the caring needs of the patients and felt like they acted against their caring values
Jerpseth et al. (2018) Norway [44]	Two university hospitals and three district hospitals	12 patients with severe COPD; 5 males, age range 63–87 years, FEV_1_% predicted NR, 6 used NIV previous year	To explore the illness experiences of older patients with late-stage COPD and to develop knowledge about how patients perceive their preferences to be taken into account in decision-making processes concerning IMV and/or NIV	Qualitative design with hermeneutic–phenomenological approach	Patients clung to the hope that the NIV treatment would help them through what the experienced respiratory crisis. The mask was tiresome, unpleasant and a bother, but also seen as symbol of hope and survival even when there was no prospect of healing. Some described “waking up” on NIV several hours or days after hospitalisation which created a sense of vulnerability. Patients were not able to remember whether anyone had ever asked them if they wanted to use NIV, nor whether they had discussed the burden versus benefit of the treatment with either their physicians or their nurses
Jones, et al. (1998), UK [50]	Home based	11 patients (all ex-smokers in severe type II respiratory failure, were electively admitted between 1991 and 1995. All were diagnosed as having COPD and were receiving maximal drug therapy); 8 males, mean age 60 (range 45–73), FEV_1_ predicted to 27 (SD 8.9) %	To test domiciliary nocturnal intermittent positive pressure ventilation (NIPPV) in patients with respiratory failure due to severe COPD	Follow up study for over two years (pre/ post)	Hospital admissions and GP consultations were halved after one year compared with the year before NIPPV and there was a sustained improvement in arterial blood gas tensions at 12 and 24 months when breathing air, despite progressive deterioration in ventilatory function. BMI did not change during the period of observation. The median survival was 920 days, with no patient dying within the first 500 days
Kuo et al. (2019) Taiwan [45]	Home, hospital	8640 patients with COPD; 69,4% males, mean age 79.97 (SD 9.87) year, FEV_1_% predicted NR	To explore and compare EOL resource use during the last six months before death between COPD and LC patients (1) comparing EOL health care resource utilization and the use of intensive and supportive procedures during the last six months of life, (2) exploring changes in the trends of intensive procedures and palliative care between 2000 and 2012, and (3) examining predictive factors of the use of intensive procedures	Retrospective cohort study	Significantly more patients with COPD (16.54%) than patients with LC (13.53%) received non-invasive MV during the last six months of their life whole
Kvangarsnes et al. (2012) Norway	Hospital; ICU	10 patients with COPD; 5 males, age range 45–85 years, FEV_1_% predicted NR	To explore patient perceptions of COPD exacerbation and the patients’ experiences of their relations with health personnel during care and treatment	Narrative inquiry	Patients’ perceptions of breathlessness were an essential theme, making them completely dependent on others regarding the mask treatment and breathing assistance. All patients had a positive experience with NIV treatment. Patients revealed stories of trust and distrust receiving NIV treatment
Landers et al. (2015) New Zealand [61]	Hospital; admitted respiratory specialist services	15 patients with severe COPD; 9 males, mean age 69.2 (SD 8.2, age range 55–89) years, mean FEV1% predicted 26.4 (SD 10)	To explore the experience of patients with advanced COPD after a life-threatening event, particularly focusing on end-of-life (EOL) issues	Grounded Theory	Some participants identified the need for acute hospital care to manage symptoms as a milestone (for example with a BiPAP in the ICU). These participants expressed confidence in the hospital to reduce their physical symptoms and related anxiety. Acute hospital care was often seen as a haven or place of security. Participants explained how such interventions (NIV) were required to keep them alive; however, the negative prognostic implication of these admissions were not explored by participants
McEvoy et al. (2009) Australia [63]	Hospital, sleep/ respiratory medicine departments and at home for the NIV long-term oxygen therapy (LTOT) group	144 patients with severe stable smoking-related COPD; 69% male, mean age 68, FEV1% predicted LTOT: 23.1 (21.4 to 24.8), NIV + LTOT: 25 (22.4 to 27.6)	To determine the effects of nocturnal non-invasive bi-level pressure support ventilation (NIV) on survival, lung function and quality of life in patients with severe hypercapnic COPD	A multicentre, open-label, RCT	NIV improved sleep quality and sleep-related hypercapnia acutely, and patients complied well with therapy (mean (SD) nightly use 4.5(3.2) h). Compared with LTOT alone, NIV (mean follow-up 2.21 years, range 0.01–5.59) showed an improvement in survival with the adjusted but not the unadjusted Cox model (adjusted hazard ratio (HR) 0.63, 95% CI 0.40 to 0.99, *p* = 0.045; unadjusted HR 0.82, 95% CI 0.53 to 1.25, *p* = NS). FEV 1.0 and PaCO2 measured at 6 and 12 months were not different between groups. Disease-specific QOL (SGRQ) at 12 months was not different between the two groups. Patients assigned to NIV + LTOT had reduced general and mental health and vigour on SF 36
Sinuff et al. (2008) Canada and US [68]	Academic or community centers	Intensivists, pulmonologists, and respiratory therapists (RTs). 104 of 183 (57%) physicians and 290 of 473 (61%) RTs participated	To determine clinicians’ attitudes to and stated use of NIV for patients with acute respiratory failure who have declined intubation and resuscitation or have chosen comfort measures only	Multi-center survey	2/3 of physicians include NIV during life support discussions with do-not-resuscitate patients at least sometimes, and 87% of RTs stated that NIV should be included in such discussions. For patients choosing comfort measures only, almost half of physicians reported including NIV as an option in their discussions at least sometimes, while fewer than half of RTs stated that these discussions should be conducted Most (> 80%) physicians use NIV and most (> 80%) RTs are asked to initiate NIV for do-not-resuscitate patients with COPD
Volpato et al. (2022) Italy [60]	Inpatients and outpatients	90 patients with severe COPD; 46 males, mean age 76.2 (SD 8.03) years, mean FEV_1_% predicted 50.7 (SD 27.0)	To analyze the impact of a brief psychological support intervention on adherence to NIV among patients with COPD	RCT	The psychological intervention was related to improvements in adherence to NIV and QOL after four to eight meetings with cognitive and behavioural therapy, with homework, during the NIV adaption compared with the control group (six sessions watching video related to COPD management). Results indicated a significant change in the QOL also over time
Windisch et al. (2005) Germany [54]	Hospital	34 patients with stable COPD and hypercapnic respiratory failure; 27 males, mean age 63.4 (SD 9.7, range 43–77),	To assess changes in blood gas levels and long-term outcome in a larger group of patients with COPD and chronic hypercapnic respiratory failure who were treated by controlled NIV aimed at achieving maximal improvement of PaCO_2_	Retrospective study	Daytime Paco_2_ during spontaneous breathing significantly decreased by 6.9 (SD 8.0) mm Hg, daytime Pao_2_ significantly increased by 5.8 (SD 9.4) mm H, FEV_1_ significantly increased by 0.14 (SD 0.16) L after 2 months of NIV. This was achieved with mean inspiratory pressures of 27.7 (SD 5.9) cm H_2_O (range, 17 to 40 cm H_2_O) at a mean respiratory rate of 20.8 (SD 2.5 breaths/min. The 2-year survival rate was 86%

Outcome measures used in the studies varied according to study aim and design (Table 4). All RCT and pre-post studies (*n* = 9) used lung and respiratory muscle function tests to measure the effect of NIV. All nine studies included spirometry; other tests varied (i.e., maximum inspiratory pressure, diaphragmatic activity). Six of the nine studies measured dyspnoea and exercise tolerance (for example, by using the 6-min walking test or Borg Dyspnoea Scales) [43, 58, 60, 69, 70, 72]. Arterial blood gases, lactate, or haematocrit were measured in eight studies [50, 58, 60, 63, 69–72], while sleep quality was measured in four studies, mostly using polysomnography [58, 63, 69, 70]. Hospital admittance was measured in five studies [50, 58, 63, 69, 70]. Seven of these nine studies used patient-reported outcome measures (PROMS), such as health-related quality of life (HRQOL) (*n* = 3) [63, 69, 72] and different measures of satisfaction, mood, or well-being (*n* = 4) [50, 60, 63, 71]. In addition, these studies applied a variety of anthropometric measurements, such as BMI, blood pressure, and SpO2. Four of the ten studies with a cross-sectional or survey design used only a few outcome measures: three included HRQOL PROMS [47, 48, 59], and one measured depression [59]. Four studies included lung and respiratory muscle function tests [40, 53, 54, 67]. Four cross-sectional studies measured different types of medical utilization, such as readmission and the number of emergency room visits [40, 42, 54, 67].. In addition, studies mapped treatment preferences, opioid use, and PC use. In addition, studies mapped treatment preferences, opioid-use, and PC use. The two cross-sectional studies, including HCPs [41, 68], both mapped attitudes, considerations, and decision-making regarding NIV treatment.

**Table 4:** Overview of the outcome measures in the included studies

Author, year, design	Patient reported outcome measures	Lung and respiratory muscle function tests	Dyspnoea and exercise tolerance	Sleep quality	Blood tests	Hospitalisation, intubation, and survival rate	Others
**Randomized controlled trial (RCT) / pre-post design:**							
Aliannejad et al. (2015) [69] Pre/post	**Health-related quality of life (HRQOL):** St George`s Respiratory Questionnaire (SGRQ)	Spirometry Diffusion capacity Oxygen saturation (during test)	VAS scale Borg dyspnoea scales (resting dyspnoea) 6-min walking test (6MWT)	Pittsburgh Sleep Quality Questionnaire (Persian)	Arterial blood gases	Number and location (intensive care unit (ICU) stays, intubations), deaths	Treatment compliance (time on non-invasive ventilation (NIV)), body mass index (BMI)
Borghi-Silva et al. (2010) [72] RCT	**HRQOL**: SGRQ	Spirometry (FEV, vital capacity) Maximum inspiratory and expiratory pressure	6MWT, Cardiopulmonary Exercise testing (treadmill) Isokinetic knee extension strength and leg fatigue test	Not reported (NR)	Lactate (during test)	NR	Oxygen saturation (SaO_2_), blood pressure, heart rate (during test)
Casanova et al. (2000) [70] RCT	NR	Spirometry Residual volume Functional residual capacity and total lung capacity	Gas exchange, The Medical Research Council and Borg scales, (3 + 6 months)	Nocturnal respiratory polysomnography	Haematocrit	Hospital admissions (primary), Intubations and mortality (3, 6 and 12 months)	Echocardiogram and cardiac function (cardiologist), neuro-psychological performance test (by psychiatrist)
Funk et al., (2011) [58] RCT	NR	Spirometry Maximum inspiratory pressure	6MWT Exercise PaCo_2_ (bicycle-test)	Polysomnography (Without NIV)	Arterial blood gases	Time to clinical worsening (escalation of mechanical ventilation) (primary)	NR
Gloeckl et al. (2019) [43] RCT (cross-over)	NR	Muscle oxygen availability (spectroscopy) Transcutaneously measured partial pressure of carbon dioxide (TcPCO2)	Levels of dyspnoea and leg fatigue at rest (a modified Borg Scale)	NR	NR	NR	SaO_2_, Heart rate
Girault et al., (1997) [71] RCT	Respiratory comfort (level of dyspnoea, well-being) (VAS scale 0–100)	Inspiratory work of breathing volume, Diaphragmatic activity	NR	NR	Blood gas tensions	NR	SaO_2_
Jones et al., (1998) [50] Follow up (pre/ post)	Patient satisfaction (self-developed questionnaire)	Spirometric parameters	NR	NR	Arterial blood gas tensions	Hospital admissions, use of general practitioner resources	Survival, BMI, compliance with NIV
McEvoy et al., 2009 [63] RCT	**HRQOL:** SF 36 & SGRQ Profile of mood states (POMS)	Spirometry	NR	Polysomnography	Arterial blood gases	Duration of hospital admittance, survival (primary)	NR
Volpato et al. (2022) [60] RCT	The Fatigue Severity Scale, **HRQOL:** EuroQol, the Hospital Anxiety and Depression Scale (HADS), the Brief Illness Perception Questionnaire, the Questionnaire on Ad-hesion to Pharma-cological and Dietetic Therapy, the Rosenberg Self-esteem Scale	Spirometry (FEV1, FVC,FEV1/FVC)	NR	NR	Arterial blood gases	NR	BMI, cognitive functions: the Addenbrooke’s Cognitive Examination Revised, the Mini-Mental State Examination scale and the Confusion Assess-ment Method
**Author, year, design**	**Patient reported outcome measures**	**Lung and respiratory muscle function tests**	**Dyspnoea and exercise tolerance**	**Sleep quality**	**Blood tests**	**Hospitalisation, intubation, and survival rate**	**Others**
**Cross sectional / survey design**							
Budweiser et al. (2005) [53] Retrospective explorative study	NR	Inspiratory capa-city	NR	NR	Diurnal and nocturnal blood gas	NR	Adverse effects or problems
Carlucci et al., (2016) [59] Prospective	The Centre for epidemiologic studies of Depression Scale, (CES-D) **HRQOL:** the Mauger Respiratory Failure questionnaire reduced form (MRF-26)	NR	NR	NR	NR	NR	NR
Duenk et al. (2017) [67] Retrospective medical record review	NR	Global Initiative for Chronic Obstructive Lung Disease (GOLD) stage and smoking history	NR	NR	NR	Readmission within 8 weeks Date of first readmission for AECOPD and date of death	NR
Fahim & Kastelik (2014) [47] Prospective observational study	COPD palliative and prognosis questionnaire (study specific), Leicester cough questionnaire (quality of life)	NR	Borg dyspnoea scale, Medical Research council (MRC) dyspnoea scale	NR	NR	NR	NR
Fu et al. (2018) [40] Retrospective observational	NR	Pulmonary function test results (FEV_1_/FVC ratio and FEV_1_%)	Heart function two-dimensional echocardiography	NR	NR	Medical utilization (number of emergency room visits, and hospitalizations) and any cardiac pulmonary resuscitation within 1 year prior to death. Number of days from each patient’s signed DNR consent until death and the number of days from physician confirmed terminal status to patient death	Additional information obtained during the last (terminal) hospital admission included whether admitted from the ER, died on the service of a pulmonologist, died in the ICU, or experienced mechanical ventilation (such as invasive and non-invasive mechanical ventilation)
Gaber et al. (2004) [48] Survey	**HRQOL**: Breathing problem-based quality-of-life questionnaire (BP-QoL)	NR	NR	NR	NR	NR	Questions on treatment preferences in hypothetical scenario
Gäbler et al. (2019) [41] Cross sectional	NR	NR	NR	NR	NR	NR	A case vignette geriatric end-stage COPD patient with acute respiratory failure, additional questions on ethical considerations and their impact on decision making
Gershon et al. (2018) [42] Repeated Cross sectional	NR	NR	NR	NR	NR	Rates of formal palliative care service use (hospital, outpatient, palliative care unit/hospice setting)	Opioid use, long-term oxygen therapy (LTOT)
Sinuff et al. (2008) [68] Multi-center Survey	NR	NR	NR	NR	NR	NR	Developed an instrument to assess the attitudes of intensivists, pulmonologists, and respiratory therapists toward the use of NIV for patients with acute respiratory failure near or at the end of life
Windisch et al. (2005) [54] Retrospective study	NR	Body plethysmography, inspiratory mouth occlusion pressure	NR	NR	Arterial blood gas tests	Duration of hospital stay, time of death	NR
**Author, year, design**	**Patient reported outcome measures**	**Lung and respiratory muscle function tests**	**Dyspnoea and exercise tolerance**	**Sleep quality**	**Blood tests**	**Hospitalisation, intubation, and survival rate**	**Others**
Cohort studies							
Budweiser et al. (2006) [55] Prospective cohort	NR	Whole-body plethysmography, Inspiratory mouth-occlusion pressure and maximum static inspiratory mouth pressure	NR	NR	Blood gas	NR	BMI
Budweiser et al. (2007) [52] Prospective cohort	NR	Spirometry body plethysmography	Exacerbation control (increased dyspnoea, cough and sputum)	NR	Blood gas (earlobe)	All-cause mortality	NR
Elliott et al. (1992) [51] Prospective Cohort	Compliance, symptoms of hypercapnia, side effects with mask, satisfaction (study specific)	Spirometry, Pulmonary func-tion test results (FEV_1_, FVC%, Total Lung Capacity (TLC)%)	NR	NR	Blood gas	NR	SaO2, BMI
Faes et al. (2018) [64] Retrospective, full population analysis	NR	NR	NR	NR	NR	Describe resource use in the last six months of life (i.e. emergency room visits, hospitalization, intensive care unit medical equipment and palliative care)	NR
Kuo et al. (2019) [45] Retrospective cohort study	NR	NR	NR	NR	NR	Numbers and costs of out-patient visits, hospitalizations, emergency room visits, intensive care unit admissions, and palliative care + haemodialysis day	COPD medications during a year

*FEV1* Forced expiratory volume in the first second, *FVC* Forced vital capacity, *FEV1/FVC* Ratio between forced expiratory volume in the first second and forced vital capacity or Tiffeneau Index, *DNR* Do not resuscitate

In the five cohort studies, two studies measured lung and respiratory muscle function using body plethysmography [52, 55], another used spirometry [51], and the same three studies also measured blood gas. Three of the cohort studies measured different types of resource use, such as the numbers of out-patient visits, palliative care and number of hospitalizations [45, 52, 64]. One study measured SaO2 and BMI [51].

The findings are presented in four thematic groupings: 1) preferences and attitudes towards the use of NIV, 2) patient participation in the decision-making process of NIV treatment, 3) conflicting results on perceived the benefits and burdens of NIV treatment, and 4) heterogenous clinical outcomes in experimental studies.

#### Preferences and attitudes towards the use of NIV

Seventeen studies reported patients’ and HCPs’ preferences and attitudes towards the use of NIV in hospital or at home [40–42, 44, 45, 47–50, 59–62, 64–68].

When a scenario-based approach was used to elicit patients’ end-of-life preferences and preferences regarding artificial ventilation and resuscitation, 40–50% of the patients chose NIV [48, 59]. Patients perceived the NIV mask as a ‘life buoy’, a symbol of survival and hope that could help them through a respiratory crisis [44, 62, 65] and as helpful even when there was no chance of cure [44]. Patients interviewed in a hospital setting believed that NIV treatment in the intensive care unit (ICU) or the use of home-based NIV was necessary to manage their symptoms, keep them alive, and prolong their life [44, 49, 61]. In one study, 75% of the home-based patients that used NIV over two years reported that they were very satisfied with the treatment [50]. An RCT found that a brief psychological intervention significantly improved patients’ acceptance of and adherence to NIV [60]. Hospital-based patients who received NIV treatment more than once felt that familiarity with the treatment made it easier to cooperate in acute situations [65]. Patients who experienced the NIV treatment as awful changed their attitudes towards it after hospital discharge and wanted NIV treatment again in the future [66]. A study that compared end-of-life care utilisation between patients with COPD and patients dying of other diseases, such as lung cancer and cardiovascular disease, found that patients with COPD were more likely to receive NIV in the last six months of life than the patient groups with lung cancer and cardiovascular disease [45, 64]. Another study found that in 2004, 1% of the patients with COPD admitted to the ICU needed NIV, while approximately 4% of the patients needed NIV in 2014 [42].

A study [41] investigating physicians’ treatment choices by using a case vignette featuring a geriatric patient with end-stage COPD and acute respiratory failure showed large differences between the choices made by the doctors. Twenty-three percent of the physicians chose NIV, while 46% chose a palliative approach without respiratory assistance. Pulmonologists and internists were more likely to choose NIV than palliative physicians or geriatricians. Increasing age of physicians was associated with not starting with NIV; no effect was found regarding educational level and years of professional experience [41]. A survey showed that the majority of RT and physicians were more likely to choose NIV for patients with COPD or cardiogenic pulmonary oedema with a do-not-resuscitate order near the end of life than for patients with underlying malignancy with a do-not-resuscitate order [68]. HCPs felt limited by the lack of clear evidence of measurable benefits of home-based NIV, such as hospital admissions and mortality, and patient selection for NIV treatment was carried out on a case-by-case basis [49]. HCPs discussed ethical challenges, such as patients’ resistance to using NIV or terminating the NIV treatment when patients were in the end-stage of COPD [65].

#### Patient participation in the decision-making process of NIV treatment

Ten studies reported patients’ and HCPs’ experiences of challenges regarding patient participation in the decision-making process of NIV treatment in hospital or at home [40, 41, 44, 46, 48, 49, 56, 57, 62, 65, 66].

Hospital-based patients who had used NIV described circumstances in which they experienced lack of control, felt vulnerable, recalled feelings of impending death, and were totally dependent on HCPs due to breathlessness, anxiety, and panic [44, 56, 62]. Moreover, patients felt that they were dependent on HCPs for survival [44]. Many wanted to take part in the decision-making process regarding NIV treatment [44, 48, 49, 56], and in one study most patients expressed that treatment such as NIV should be discussed with all patients [48]. Patients deemed that demonstration of NIV was more helpful than the use of a photograph of an NIV machine in use on a patient and increased patients’ willingness to use NIV [46].

Patients expressed that they had been included by HCPs in such decision-making to varying degrees [44, 48, 49, 56], and they often felt that they had no choice other than to use NIV because physicians recommended it or because their deteriorating health condition demanded it [44, 49]. Other patients expressed that the HCPs should make the treatment decisions as they believed that physicians were doing their best and acted in their best interest [44, 49, 56, 62, 65].

Although hospitalised patients handed over the decision-making to HCPs, they still interacted with nurses and participated actively in what was happening to them [56]. The medical records of patients who died in hospital showed that patients with an early do-not-resuscitate order used less IMV, used NIV more often, died less often in the ICU, and had a shorter length of stay in hospital than those with a late do-not-resuscitate order [40].

HCPs pointed out the importance of using time to communicate the aim of home-based NIV to the patients to facilitate the patients’ choices and enhance adherence. In this way, the HCPs underpinned that the decision to use home-based NIV ultimately lay with the patients [49]. Responding to a case vignette, around 40% of the physicians commented that they would have wanted to know if the patient had any sort of precautionary directive [41]. Nurses perceived that they were part of a treatment culture that focused more on medical treatment than creating optimal end-of-life care and that the only measures offered to support patients’ capacity to breath were NIV or IMV. According to these nurses, the interdisciplinary meetings were short and mostly involved information about patients’ objective symptoms regarding their lungs and laboratory tests. In this context, there was no room to discuss patients’ participation in treatment decisions, undertake care planning, or pay attention to the patients’ QOL, suffering, or functional status [57].

#### Conflicting and divergent results on the perceived benefits and burdens of NIV treatment

Fifteen studies reported on conflicting and divergent results on the perceived benefits and burdens in NIV treatment in hospital and at home from the perspective of patients, relatives, and HCPs [43, 44, 49–51, 56, 58, 62, 63, 65, 66, 68–72] (the PROMS are described in Table 4).

Patients spoke of the benefits of NIV treatment, such as easing chest pressure [56], and experienced the spontaneous breathing mode as more comfortable than the assist-control ventilation mode [71]. Patients reported that they complied well with nocturnal NIV plus LTOT [63]. Several burdens were described for NIV treatment, such as the NIV mask being a bother, unpleasant, and tiresome as it was often too tight, caused facial sores, dryness in the mouth, and claustrophobia with feelings of suffocation and made it difficult to communicate [44, 49, 50, 62, 65, 66]. Patients were afraid of being alone with the ventilator and underlined the importance of knowing that they were not alone [65, 66]. A few patients did not tolerate the NIV treatment within the first weeks and expressed that the experienced pressure of the NIV was too high [70]. Some patients withdrew from a study as they were not able to sleep using NIV [51].

Studies reported conflicting results on whether the use of NIV improved HRQOL and dyspnoea. Patients using home-based NIV reported improved QOL and well-being, reduction of dyspnoea, and having more energy to carry out activities in everyday life. Some expressed that they got their old life back while using home-based NIV [49]. The relatives had similar thoughts [49]. In a pre-post study, home-based NIV improved HRQOL and the symptom and total St George Respiratory Questionnaire (SGRQ) scores were improved [69]. An RCT that consisted of a 6-week physical training program (treadmill walking) for patients using NIV or supplemental oxygen found that the HRQOL measured using the SGRQ improved in patients in both the NIV and oxygen group, while the total and activity SGRQ score significantly improved in only the NIV group [72]. An RCT that determined the effect of withdrawal of NIV in patients who remained hypercapnic after acute respiratory failure found no significant differences in HRQOL using the SGRQ three months after randomisation between the NIV group and those who stopped using NIV. It was not possible to analyse long-term effects on HRQOL due to a small remaining sample [58]. Furthermore, an RCT found that patients who used nocturnal NIV plus LTOT reported less vigour and more confusion and bewilderment (measured with SF 36 and the Profiles of Mood States) than the controls (LTOT) [63]. Another study found no change in QOL in patients using nocturnal home-based NIV [51]. Patients using nocturnal NIV plus LTOT and NIV or NIV only reported significantly improved sleep quality [50, 63, 69].

Additionally, in another RCT patients in the nocturnal NIV plus LTOT group reported significant improvement of dyspnoea on both the Medical Research Council scale and the Borg Scale by the third month compared with the control group (LTOT). By the sixth month, improvement of dyspnoea in favour of the nocturnal NIV was evident on the Borg Scale [70]. In a follow-up study stretching over two years, the patients using NIV reported no improvement in dyspnoea [50, 69]. Patients using NIV while participating in physical training reported greater improvement than the supplemental oxygen group regarding dyspnoea [72] and showed a lower increase in exertional dyspnoea in a cross-over trial [43].

In a survey involving physicians, more than 50% reported that for patients with a do-not-resuscitate order, NIV added to the alleviation of dyspnoea provided by anxiolytics and analgesics and that NIV facilitated communication with HCPs and relatives. Regarding patients with a comfort-measures-only order, a minority of RT and physicians agreed that NIV provided either of these two benefits [68]. In another study, HCPs reported that communicating with patients using NIV was difficult due to the tight mask and the patients’ condition [65].

Results from a qualitative study reported that HCPs struggled to balance the potential of NIV to reduce QOL with its uncertain clinical effectiveness [49].

#### Heterogenous clinical outcomes in experimental studies

Eleven studies reported on heterogenous clinical outcomes in experimental studies regarding the use of NIV [43, 50, 52–55, 58, 63, 69, 70, 72] (the clinical outcomes are shown in Table 4).

A randomised cross-over trial found that patients using NIV plus oxygen showed a 39% increase in cycle endurance time and reduced exercise-induced hypercapnia compared with the control group (oxygen only) [43]. In another RCT, after a physical training programme (treadmill walking) the patients in the NIV group significantly increased their respiratory muscle strength, peripheral muscle strength, and 6-min walking distance and showed decreased leg fatigue and reduced lactate/speed ratio compared with controls (oxygen) [72].

An RCT found that patients who were hypercapnic after acute respiratory failure who needed NIV and stopped using it had a higher chance of clinical worsening (escalation of IMV) than those who continued to use NIV. The 6-min walking distance increased in the NIV group, while it decreased in those who stopped using NIV [58]. A pre–post study found that the number of hospital admissions and exacerbations was significantly reduced in patients with severe mustard airway disease using NIV [69].

In a follow-up study of patients who used nocturnal home-based NIV, the use of general practitioners and hospitalisations halved after one year compared with the year before NIV. The median survival was 920 days, and no patients died during the first 500 days [50]. Studies also showed that nocturnal NIV significantly reduced paCO_2_ [50–54]. A retrospective study found that hypercapnia improved significantly during nocturnal NIV in hospitalised stabile patents with hypercapnic COPD using respiratory pressures with a mean of 28 cm H_2_O [54]. An RCT showed improved survival for patients using nocturnal NIV plus LTOT compared with controls and found improved sleep-related hypercapnia in favour of the NIV groups compared with controls [63].

Another RCT that evaluated the one-year efficacy of NIV plus LTOT found that the one-year survival and number of acute COPD exacerbations were similar in the NIV plus oxygen group and the control group (97% used LTOT). The number of hospitalisations decreased at three months in the NIV plus LTOT group compared to the control group; however, this effect was not seen at six months or at one year [70]. A study found that the mortality rate in patients with hypercapnic respiratory failure using home-based NIV was 44.7% with one-year, two-year, and five-year survival rates of 84.0%, 65.3%, and 26.4%, respectively [52].

A study evaluated the prevalence of malnutrition (body mass index < 20 kg/m^2^) and the longitudinal changes in nutritional status in patients undergoing NIV. There was a significant weight gain among the malnourished patients after the implementation of NIV. The weight gain was not correlated with improvements in lung function or blood-gas values [55].

### Consultation exercise

The nurses expressed great recognition of the presented findings and their relevance for clinical practice and supplemented the review findings with reflections regarding their own experiences of administering NIV treatment to these patients. These experiences concerned lack of information on how to use home-based NIV to enhance QOL; tailoring the use of NIV to prevent complications; professional competency regarding NIV as palliative treatment; and ethical challenges using NIV as a palliative measure. Sharing these preliminary findings with peers increased the relevance of our findings for future research and COPD practice. Additionally, we believe that the consultation exercise served as a mechanism to keep us informed about evolving trends in NIV treatment. It would have alerted us to any changing priorities within the field that might not have been covered by the included studies.

## Discussion

Our findings suggest that patients perceived NIV as a ‘life buoy’ to keep them alive. Even though HCPs would choose and use NIV in patients with severe COPD, they may not perceive NIV as a palliative measure. Many patients wanted to take part in the decision-making process regarding NIV treatment but expressed being included to varying degrees by HCPs in such decision-making. Furthermore, there were conflicting findings regarding the perceived benefits and burdens of NIV treatment and diverse heterogeneous clinical outcomes in experimental studies.

Our review suggest that many patients seemed to be satisfied with NIV treatment and perceived it as a way to keep them alive, prolong their lives, and alleviate symptoms. This preference for and attitude towards NIV may be due to patients’ lack of knowledge regarding diagnosis, prognosis, and treatment as well as the absence of discussions with HCPs about PC [2, 73]. Patients often want to focus on staying alive rather than talk about end of life [74] and may be willing to undergo life-saving treatment despite discomforts with the expectation that they will recover [75, 76]. Many patients may not view NIV as a burdensome intervention [76]. Furthermore, dyspnoea is a frequent and tiring symptom that is challenging to alleviate [10, 11], and patients may have experienced that NIV treatment provided adequate alleviation of this demanding symptom.

Our findings were inconclusive as to whether HCPs perceived NIV as a palliative measure. Traditionally, PC for patients with COPD has been associated with care of the dying, and the prognostic uncertainty makes it challenging for HCPs to identify when PC should be introduced [9]. The literature suggests that NIV can also be used when intubation is not an option to prolong life or to alleviate dyspnoea and provide comfort measures only [20]. However, NIV at the end of life is controversial and has been portrayed both as a measure to improve QOL and dyspnoea and provide comfort and as a futile treatment that may prolong the dying process and increase suffering [21, 22]. A guideline suggests that NIV could be used in a palliative setting if it does not have negative consequences, such as mask discomfort or prolonging the dying process. HCP training is a prerequisite for NIV to be used as a palliative measure [77]. Furthermore, careful patient selection and setting goals in advance for such treatment, a timeframe for revaluations, and criteria for success or failure are important [77, 78].

Our findings show that patient participation in the decision-making process regarding NIV may be challenging: some patients want to participate, while others would prefer physicians to act in their best interest. Autonomy and patient participation are significant to enable patients to live a good life based on their needs and preferences [79]. Our findings describe that patients felt they were included to varying degrees in the decision-making process or that they had no choice other than to use NIV since HCPs recommended it or due to their deteriorating health [80]. Patients may not consider future treatment, be reluctant to make binding decisions regarding future care [81], or feel that such discussions are pointless as the future seems beyond their control due to their disease [75]. The timing of such discussions needs to be tailored, but they should be carried out early in the disease trajectory when patients are in a stable phase of the disease by a physician who has a trusting relationship with them [82]. However, HCPs may be reluctant to discuss PC and future treatment since they mainly focus on curative treatments or may be concerned about destroying patients’ hope [2, 9].

Our findings show that patients experience burdens regarding NIV treatment, such as the negative consequences of wearing a mask, and that HCPs were concerned that NIV could reduce patient QOL. In a hospital-based study, patients at all stages of COPD and acute respiratory failure undergoing NIV treatment described ambivalent feelings regarding NIV due to concerns over whether the struggle and discomfort experienced when using the mask and ventilation were worthwhile. For some patients, the discomfort caused by the mask and ventilation was perceived as minor compared with the feeling of dyspnoea [83]. Dyspnoea and HRQOL are recognised as among the most important patient-centred outcomes in patients with COPD [84]. However, our findings showed conflicting results as to whether NIV improved these outcomes. This could be due to the heterogeneity of these studies, such as different study designs, outcome measures, data collection points, and duration of NIV. In contrast to a randomised feasibility study that suggested that NIV was more effective than oxygen in reducing dyspnoea in patients with cancer (life expectancy less than six months) [85], none of the studies in our review were designed to assess the acceptability of NIV as the only palliative measure.

Some of the studies in our review suggested that NIV may reduce the number of hospitalisations and COPD exacerbation as well as increase exercise capacity. Many patients want to spend as much time as possible at home and even want to die there [86] as patients may feel more comfortable and in control at home than in the hospital [87]. However, patients with COPD are more likely to die in hospital or nursing homes than patients with lung cancer [2, 86]. Relatives reported that during the last year of life patients with COPD were afraid of being left alone and of dying due to dyspnoea, anxiety, and panic [88]. Such fear may contribute to hospitalisation. Furthermore, exercise capacity is recognised as another important outcome in patients with COPD [84]. By alleviating dyspnoea and increasing exercise capacity, home-based NIV may enable patients to spend more time at home and participate in a few meaningful activities. Moreover, the use of telehealth may support and enable these patients to spend more time at home. A systematic mixed-studies review suggests that home-based patients with PC needs experience telehealth as a potential support system that could enable them to remain at home and that self-reporting could provide HCPs with information about patients’ symptoms and circumstances that can be used to tailor care [89].

Based on the findings from this review, we recommend that future studies should be designed to examine the acceptability, experiences with, and effectiveness of the use of NIV as a palliative measure to alleviate dyspnoea and improve QOL and other PROMS. As diverse patient-centred outcomes and clinical outcomes are reported, future studies should determine which are most important. Studies should also explore how palliative home-based NIV treatment and dependence on masks impact patients’ and relatives’ daily life and QOL. Due to challenges to including patients in the decision-making process of NIV treatment, patients, relatives, and HCPs should be included in the co-design of interventions to enhance patient health literacy and participation. The relatives’ voice was only included in two studies, and their experiences of providing informal care at home and experience with NIV at end of life should be investigated. Studies that examined HCPs’ perspectives and experiences with the use of NIV were mostly limited to physicians. However, nurses administer NIV treatment and observe the effects and adverse events of the treatment. Consequently, studies on nurse training and competence in NIV and studies with a multidisciplinary approach are warranted. We emphasize that by doing the consultation exercise including respiratory and critical care nurses contributed to nuance and validate the review findings, and also highlighted that this topic is highly relevant within nursing care.

### Limitations

Synonyms of both PC and NIV may exist that we were unable to identify and include in our database search strategy. In addition, as our review had some language limitations, there may be studies that we were unable to identify. Another limitation may be that in the included studies the patients may not have been regarded as having PC needs and NIV treatment may have had a curative or restorative intention rather than a palliative intention.

We chose to include nurses from the hospital setting in the consultation exercise as they care for patients using NIV around the clock, and their voice was included in very few studies. Nurses have extensive experience and competence regarding administering NIV to patients with COPD. However, a limitation may be that we did not include nurses from homecare setting, physicians as they have the ultimate responsibility for medical decision-making or physiotherapist or respiratory therapist as they also have a crucial role in NIV to these patients. Including multidisciplinary HCPs, as well as patients and relatives could have provided other type of feedback regarding the clinical relevance of our findings. The sample size of the consultation exercise may be a limitation. However, the participants had extensive experience regarding the use of NIV to these patients and provided rich input regarding the review findings.

We conducted the scoping review in line with an acknowledged methodological framework and the PRISMA extension for Scoping Reviews checklist and did not appraise the methodological quality of the included studies and synthesise the data. A potential source for heterogeneity in our scoping review is studies included from different countries as there are differences in cultural backgrounds, healthcare services, resources available, healthcare guidelines, policies and approaches. Furthermore, we also included older studies as we wanted to describe the entire range of studies relevant for our research question. Thus, our findings and how the findings can inform clinical practice (e.g. the effectiveness of NIV) need to be interpreted with caution.

## Conclusion

Patients with COPD with PC needs seem to view NIV as a treatment that can keep them alive rather than as a palliative measure. HCPs use NIV in these patients but may not consider it a palliative measure, suggesting that NIV may be introduced with a curative or restorative intention rather than a palliative one. The included studies mostly mirror the physicians’ views on NIV as a treatment option and its physiological outcomes. There is a knowledge gap related to competence development in the palliative use of NIV and to the experience of other stakeholders, especially homecare nurses. Future studies should be designed to explore and evaluate the use of palliative NIV treatment more clearly. As some patients may be reluctant to participate, or may not be included, in the decision-making process regarding NIV, measures for enhanced communication and information about NIV and the inclusion of patients in such processes early in the disease trajectory are warranted. The included studies reported the conflicting benefits and burdens of NIV treatment and heterogeneous clinical outcomes. Consequently, future studies should investigate which outcomes are the most important, effective, and relevant for optimal treatment when NIV is used in patients with COPD with PC needs.

### Supplementary Information

**Additional file 1.** Deviations from the published protocol.

**Additional file 2.** Search strategy all databases.

## Data Availability

The data collected and thematically grouped are publicly available, as were all the data collected from published studies in peer-reviewed scientific papers.

## Abbreviations

AMED: Allied and Complementary Medicine
CINAHL: Cumulative Index to Nursing and Allied Health Literature
COPD: Chronic obstructive pulmonary disease
Embase: Excerpta Medica Database
HCPs: Healthcare professionals
HRQOL: Health-related quality of life
IMV: Invasive mechanical ventilation
LTOT: Long-term oxygen treatment
MEDLINE: Medical Literature Analysis and Retrieval System Online
PC: Palliative care
PEDro: Physiotherapy Evidence Database
PsycInfo: Psychological Information Database
NIV: Non-invasive ventilation
QOL: Quality of life
