# Supplementary material for: Non-invasive ventilation in the care of patients with chronic obstructive pulmonary disease with palliative care needs: a scoping review
Source: BMC Palliat Care. 2024 Jan 29;23:27. doi: 10.1186/s12904-024-01365-y (PMC10823671; doi:10.1186/s12904-024-01365-y)
Supplement: Supplementary file 1 — Additional file 1. Deviations from the published protocol. [file 12904_2024_1365_MOESM1_ESM.pdf]

Appendix 1 Deviations from the published protocol

Planned approach	Deviations from protocol
Title: Non-invasive ventilation in the palliative care of patients with chronic obstructive pulmonary disease: A scoping review	Title: Non-invasive ventilation in the care of patients with chronic obstructive pulmonary disease with palliative care needs: A scoping review
The aim of this scoping review was to systematically map published studies on the use of NIV in the palliative care (PC) of patients with COPD, including the perspectives and experiences of patients, families, and healthcare professionals (HCPs).	The aim of this scoping review was to systematically map published studies on the use of NIV in patients with COPD with palliative care needs, including the perspectives and experiences of patients, families, and healthcare professionals (HCPs).
Seven pairs of authors will independently assess whether titles and abstracts and then full-text papers meet the eligibility criteria. In cases of disagreement, a third author (SAS, KH, HA or MHL) will conduct an independent assessment, and a final decision will be made based on consensus among the three authors.	Covidence ensured that two authors independently assessed whether titles and abstracts and then full-text papers meet the inclusion criteria (Table 1). In cases of disagreement, two authors (SAS, & MHL) performed an independent assessment, and a final decision was based on consensus among these two authors.
The first, second, third, and last author analysed the data, while all the authors discussed the emerging patterns and agreed upon the final thematic grouping.	The first, and last author analysed the data, while all the authors discussed the emerging patterns and agreed upon the final thematic grouping.
The results will be discussed with an advisory board consisting of six to eight nurses and physicians from respiratory and intensive care units at a university and a community hospital who work with patients with COPD and NIV. If possible, service users from a COPD patient organisation will also join the advisory board.	We chose to include only nurses in the consultation exercise as they care for patients using NIV around the clock, and their voice was included in very few studies.
We will facilitate a 2–3 hours workshop. In the workshop the first, second and last author of the scoping review will attend, presenting relevant preliminary results from stage 5 of the review and discuss the importance and novelty for clinical practice with the advisory board members.	Each workshop lasted for 1 hour. The first and second authors attended.
