# Supplementary material for: Non-invasive ventilation in the care of patients with chronic obstructive pulmonary disease with palliative care needs: a scoping review
Source: BMC Palliat Care. 2024 Jan 29;23:27. doi: 10.1186/s12904-024-01365-y (PMC10823671; doi:10.1186/s12904-024-01365-y)
Supplement: Supplementary file 2 — Additional file 2. Search strategy all databases. [file 12904_2024_1365_MOESM2_ESM.pdf]

Appendix 2 Search strategy all databases

For each database, the search strategy was built with three elements. Each element consists of a combination of subject headings from the actual databases' thesaurus, and textwords describing the actual element. The first element searches for Palliative care, the second element for chronic obstructive pulmonary disease, and the third element searches for non-invasive ventilation treatment. The search words within an element are combined with the boolean operator OR, and then the results from each element were combined with the boolean operator AND. Limiters for language were applied, and if possible, letters, editorials, commentaries and conference abstracts were removed.

Database(s): Ovid MEDLINE(R) and Epub Ahead of Print, In-Process & Other Non-Indexed Citations and Daily 1946 to November 14, 2022

#	**Searches**
1	Palliative Care/
2	"Hospice and Palliative Care Nursing"/
3	exp Terminal Care/
4	Palliative Medicine/
5	exp Advance Care Planning/
6	"Right to Die"/
7	Terminally ill/
8	(palliative or palliate* or palliating).tw,kf.
9	(dying or (right adj2 die) or (die adj2 dignity)).tw,kf.
10	"supporti* care".tw,kf.
11	((terminal* or "end stage*" or endstage* or "advanced stage*" or "late stage*") adj3 (disease* or ill* or care* or caring or treatment* or period* or nurs* or patient*)).tw,kf.
12	(eol or "end of life").tw,kf.
13	(("life limiting" or "life threatening") adj3 (disease* or condition* or illness*)).tw,kf.
14	Resuscitation Orders/ or ("do not" adj3 (intubat* or resuscitat*)).tw,kf.
15	(DNR or DNAR or DNI).tw,kf.
16	"comfort measure*".tw,kf.
17	(advance*1 adj3 (plan*1 or planning or directive*)).tw,kf.
18	hospice*.tw,kf.
19	or/1-18
20	exp Pulmonary Disease, Chronic Obstructive/
21	(COPD or COAD or COBD or AECB).tw,kf.
22	((chronic or "long-term" or longterm or lifelong or "life-long" or permanent or persisting) adj3 (pulmonary or lung*1 or airway* or airflow*)).tw,kf.
23	((chronic or "long-term" or longterm or lifelong or "life-long" or permanent or persisting) adj3 (Bronchitis or bronchitides)).tw,kf.
24	((pulmonary or focal or panacinar or panlobular) adj3 emphysema*).tw,kf.
25	alpha 1-Antitrypsin Deficiency/
26	(antitrypsin adj3 deficienc*).tw,kf.
27	or/20-26

28	Noninvasive Ventilation/
29	Respiration, Artificial/
30	exp Positive-Pressure Respiration/
31	exp Ventilators, Mechanical/
32	(NIV or ((non-invasive or noninvasive) adj3 ventilat*)).tw,kf.
33	(CPAP or BIPAP or BPAP or NIPPV or NPPV or NCPAP).tw,kf.
34	(nasal adj2 ventilat*).tw,kf.
35	((mechanical or pulmonary) adj2 ventilator*).tw,kf.
36	(positive adj3 (airway* adj3 pressure)).tw,kf.
37	(positive adj3 (pressure adj3 (ventilation* or respiration* or expiratory))).tw,kf.
38	or/28-37
39	19 and 27
40	38 and 39
41	limit 40 to (comment or editorial or letter)
42	40 not 41
43	limit 42 to (danish or english or german or norwegian or spanish or swedish)

Database(s): Embase 1974 to 2022 November 14

#	**Searches**
1	palliative therapy/
2	palliative nursing/
3	exp terminal care/
4	right to die/
5	exp terminally ill patient/
6	(palliative or palliate* or palliating).tw,kw.
7	(dying or (right adj2 die) or (die adj2 dignity)).tw,kw.
8	"supporti* care".tw,kw.
9	((terminal* or "end stage*" or endstage* or "advanced stage*" or "late stage*") adj3 (disease* or ill* or care* or caring or treatment* or period* or nurs* or patient*)).tw,kw.
10	(eol or "end of life").tw,kw.
11	(("life limiting" or "life threatening") adj3 (disease* or condition* or illness*)).tw,kw.
12	("do not" adj3 (intubat* or resuscitat*)).tw,kw.
13	(DNR or DNAR or DNI).tw,kw.
14	"comfort measure*".tw,kw.
15	(advance*1 adj3 (plan*1 or planning or directive*)).tw,kw.
16	hospice*.tw,kw.
17	1 or 2 or 3 or 4 or 5 or 6 or 7 or 8 or 9 or 10 or 11 or 12 or 13 or 14 or 15 or 16
18	chronic obstructive lung disease/
19	asthma-chronic obstructive pulmonary disease overlap syndrome/
20	chronic bronchitis/
21	lung emphysema/
22	(COPD or COAD or COBD or AECB).tw,kw.
23	((chronic or "long-term" or longterm or lifelong or "life-long" or permanent or persisting) adj3 (pulmonary or lung*1 or airway* or airflow*)).tw,kw.
24	((chronic or "long-term" or longterm or lifelong or "life-long" or permanent or persisting) adj3 (Bronchitis or bronchitides)).tw,kw.

25	((pulmonary or focal or panacinar or panlobular) adj3 emphysema*).tw,kw.
26	alpha 1 antitrypsin deficiency/
27	(antitrypsin adj3 deficienc*).tw,kw.
28	18 or 19 or 20 or 21 or 22 or 23 or 24 or 25 or 26 or 27
29	noninvasive ventilation/
30	artificial ventilation/
31	positive end expiratory pressure/
32	intermittent positive pressure ventilation/
33	mechanical ventilator/
34	bipap device/ or continuous ventilator/ or cpap device/ or intermittent positive pressure breathing machine/ or negative pressure ventilator/ or positive end expiratory pressure ventilator/
35	(NIV or ((non-invasive or noninvasive) adj3 ventilat*)).tw,kw.
36	(CPAP or BIPAP or BPAP or NIPPV or NPPV or NCPAP).tw,kw.
37	(nasal adj2 ventilat*).tw,kw.
38	((mechanical or pulmonary) adj2 ventilator*).tw,kw.
39	(positive adj3 (airway* adj3 pressure)).tw,kw.
40	(positive adj3 (pressure adj3 (ventilation* or respiration* or expiratory))).tw,kw.
41	29 or 30 or 31 or 32 or 33 or 34 or 35 or 36 or 37 or 38 or 39 or 40
42	17 and 28 and 41
43	limit 42 to (editorial or letter)
44	42 not 43
45	limit 44 to (conference abstract or conference paper or "conference review")
46	44 not 45
47	limit 46 to (danish or english or german or norwegian or spanish or swedish)

Database(s): APA PsycInfo 1806 to November Week 2 2022

#	**Searches**
1	palliative care/
2	hospice/
3	advance directives/
4	terminally ill patients/
5	(palliative or palliate* or palliating).tw.
6	(dying or (right adj2 die) or (die adj2 dignity)).tw.
7	"supporti* care".tw.
8	((terminal* or "end stage*" or endstage* or "advanced stage*" or "late stage*") adj3 (disease* or ill* or care* or caring or treatment* or period* or nurs* or patient*)).tw.
9	(eol or "end of life").tw.
10	(("life limiting" or "life threatening") adj3 (disease* or condition* or illness*)).tw.
11	("do not" adj3 (intubat* or resuscitat*)).tw.
12	(DNR or DNAR or DNI).tw.
13	"comfort measure*".tw.
14	(advance*1 adj3 (plan*1 or planning or directive*)).tw.
15	hospice*.tw.
16	1 or 2 or 3 or 4 or 5 or 6 or 7 or 8 or 9 or 10 or 11 or 12 or 13 or 14 or 15
17	exp chronic obstructive pulmonary disease/

18	(COPD or COAD or COBD or AECB).tw.
19	((chronic or "long-term" or longterm or lifelong or "life-long" or permanent or persisting) adj3 (pulmonary or lung*1 or airway* or airflow*)).tw.
20	((chronic or "long-term" or longterm or lifelong or "life-long" or permanent or persisting) adj3 (Bronchitis or bronchitides)).tw.
21	((pulmonary or focal or panacinar or panlobular) adj3 emphysema*).tw.
22	(antitrypsin adj3 deficienc*).tw.
23	17 or 18 or 19 or 20 or 21 or 22
24	artificial respiration/
25	(NIV or ((non-invasive or noninvasive) adj3 ventilat*)).tw.
26	(CPAP or BIPAP or BPAP or NIPPV or NPPV or NCPAP).tw.
27	(nasal adj2 ventilat*).tw.
28	((mechanical or pulmonary) adj2 ventilator*).tw.
29	(positive adj3 (airway* adj3 pressure)).tw.
30	(positive adj3 (pressure adj3 (ventilation* or respiration* or expiratory))).tw.
31	24 or 25 or 26 or 27 or 28 or 29 or 30
32	16 and 23 and 31
33	limit 32 to (danish or english or german or norwegian or spanish or swedish)
34	limit 33 to ("comment/reply" or editorial or letter)
35	33 not 34

Database(s): AMED (Allied and Complementary Medicine) 1985 to November 2022

#	**Searches**
1	palliative care/
2	exp terminal care/
3	palliative medicine/
4	advance directives/
5	right to die/
6	terminal illness/
7	(palliative or palliate* or palliating).tw.
8	(dying or (right adj2 die) or (die adj2 dignity)).tw.
9	"supporti* care".tw.
10	((terminal* or "end stage*" or endstage* or "advanced stage*" or "late stage*") adj3 (disease* or ill* or care* or caring or treatment* or period* or nurs* or patient*)).tw.
11	(eol or "end of life").tw.
12	(("life limiting" or "life threatening") adj3 (disease* or condition* or illness*)).tw.
13	resuscitation orders/ or ("do not" adj3 (intubat* or resuscitat*)).tw.
14	(DNR or DNAR or DNI).tw.
15	"comfort measure*".tw.
16	(advance*1 adj3 (plan*1 or planning or directive*)).tw.
17	hospice*.tw.
18	1 or 2 or 3 or 4 or 5 or 6 or 7 or 8 or 9 or 10 or 11 or 12 or 13 or 14 or 15 or 16 or 17
19	exp pulmonary disease chronic obstructive/
20	(COPD or COAD or COBD or AECB).tw.
21	((chronic or "long-term" or longterm or lifelong or "life-long" or permanent or persisting) adj3 (pulmonary or lung*1 or airway* or airflow*)).tw.

22	((chronic or "long-term" or longterm or lifelong or "life-long" or permanent or persisting) adj3 (Bronchitis or bronchitides)).tw.
23	((pulmonary or focal or panacinar or panlobular) adj3 emphysema*).tw.
24	(antitrypsin adj3 deficienc*).tw.
25	19 or 20 or 21 or 22 or 23 or 24
26	respiration artificial/
27	exp positive pressure respiration/
28	ventilators mechanical/
29	(NIV or ((non-invasive or noninvasive) adj3 ventilat*)).tw.
30	(CPAP or BIPAP or BPAP or NIPPV or NPPV or NCPAP).tw.
31	(nasal adj2 ventilat*).tw.
32	((mechanical or pulmonary) adj2 ventilator*).tw.
33	(positive adj3 (airway* adj3 pressure)).tw.
34	(positive adj3 (pressure adj3 (ventilation* or respiration* or expiratory))).tw.
35	26 or 27 or 28 or 29 or 30 or 31 or 32 or 33 or 34
36	18 and 25 and 35
37	limit 36 to (commentary or conference or congress or congress proceedings or editorial or letter)
38	36 not 37
39	limit 38 to (danish or english or german or norwegian or spanish or swedish)

CINAHL with Full Text

S39	S19 AND S27 AND S37<br>Limiters - Language: Danish, English, German, Norwegian, Spanish, Swedish
S38	S19 AND S27 AND S37
S37	S28 OR S29 OR S30 OR S31 OR S32 OR S33 OR S34 OR S35 OR S36
S36	(positive N2 (pressure N2 (ventilation* or respiration* or expiratory)))
S35	(positive N2 (airway* N2 pressure))
S34	((mechanical or pulmonary) N1 ventilator*)
S33	(nasal N1 ventilat*)
S32	(CPAP or BIPAP or BPAP or NIPPV or NPPV or NCPAP)
S31	(NIV or ((non-invasive or noninvasive) N2 ventilat*))
S30	(MH "Ventilators, Mechanical")
S29	(MH "Positive Pressure Ventilation+")
S28	(MH "Respiration, Artificial")
S27	S20 or S21 or S22 or S23 or S24 or S25 or S26
S26	(antitrypsin N2 deficienc*)
S25	(MH "Alpha 1-Antitrypsin Deficiency")
S24	((pulmonary or focal or panacinar or panlobular) N2 emphysema*)
S23	((chronic or "long-term" or longterm or lifelong or "life-long" or permanent or persisting) N2 (Bronchitis or bronchitides))
S22	((chronic or "long-term" or longterm or lifelong or "life-long" or permanent or persisting) N2 (pulmonary or lung or lungs or airway or airways or airflow*))
S21	(COPD or COAD or COBD or AECB)
S20	(MH "Pulmonary Disease, Chronic Obstructive+")

S19	S1 OR S2 OR S3 OR S4 OR S5 OR S6 OR S7 OR S8 OR S9 OR S10 OR S11 OR S12 OR S13 OR S14 OR S15 OR S16 OR S17 OR S18
S18	hospice*
S17	(advance* N2 (plan or plans or planning or directive*))
S16	"comfort measure*"
S15	(DNR or DNAR or DNI)
S14	(MH "Resuscitation Orders") or ("do not" N2 (intubat* or resuscitat*))
S13	(("life limiting" or "life threatening") N2 (disease* or condition* or illness*))
S12	(eol or "end of life")
S11	((terminal* or "end stage*" or endstage* or "advanced stage*" or "late stage*") N2 (disease* or ill* or care* or caring or treatment* or period* or nurs* or patient*))
S10	"supporti* care"
S9	(dying or (right N1 die) or (die N1 dignity))
S8	(palliative or palliate* or palliating)
S7	(MH "Terminally Ill Patients+")
S6	(MH "Right to Die")
S5	(MH "Advance Care Planning")
S4	(MH "Hospice Care")
S3	(MH "Terminal Care+")
S2	(MH "Hospice and Palliative Nursing")
S1	(MH "Palliative Care")

PEDro (Physiotherapy Evidence Database)
(TIAB: Pallia* AND Topic: chronic respiratory disease) OR (TIAB: NIV AND Topic: chronic respiratory disease)
